# Highly Stretchable and Flexible Graphene/ITO Hybrid Transparent Electrode

**DOI:** 10.1186/s11671-016-1323-y

**Published:** 2016-02-27

**Authors:** Juhua Liu, Yaohua Yi, Yihua Zhou, Huafei Cai

**Affiliations:** School of Printing and Packaging, Wuhan University, Wuhan, Hubei 430079 People’s Republic of China; School of Electronic Information, Wuhan University, Wuhan, Hubei 430079 People’s Republic of China

**Keywords:** Flexible transparent electrode, Composite materials, Graphene, Electromechanical properties

## Abstract

The flexible hybrid transparent electrode was prepared by a two-step process: graphene film was firstly grown on Cu foil by modified thermal chemical vapor deposition (CVD) and then transferred onto indium tin oxide (ITO) electrode on the polyethylene terephthalate (PET) substrate. The quality of the graphene is characterized by various analytic techniques, including the AFM, SEM, TEM, and Raman spectroscopy. The gradient flux was found to be beneficial to decrease defect. The thickness, morphology, light transmittance, and electromechanical properties of three conductive electrodes were investigated and compared. The outcomes show that the hybrid electrode could resist mechanical force and the results are better than original ITO electrode. It may be a potential trend to apply the graphene to other conducts in the flexible transparent conductive field.

## Background

A new generation of flexible devices has been extensively studied for electronics, optoelectronics, and energy harvesting applications [1–4]. The key component for such devices is a flexible and stretchable electrode, which is able to maintain original electrical properties after bending or stretching process. Indium tin oxide (ITO) has been the industrial standard for transparent electrodes in traditional optoelectronic devices [5]. However, ITO is difficult to apply in flexible devices because of its brittleness [6, 7]. To fulfill the growing requirements of flexible and transparent electrodes, several alternative materials have been developed recently. For example, conducting polymers (specifically poly (3, 4-ethylenedioxythiophene) poly (styrenesulfonate) (PEDOT: PSS)) [8, 9], metallic nanowires [10, 11], and carbon nanotubes [12] have attracted extensive attention and employed to utilize as flexible electrode materials. But their environmental instability, surface roughness, and surface uniformity hinder the widespread applications [13–15].

Recently, graphene becomes a novel kind of two-dimensional (2D) carbon allotrope with a unique band structure, which shows outstanding thermal, mechanical, and electrical properties [16]. Besides, it is well known that graphene has high transmittance and high electron mobility [15, 16]. There have been efforts to utilize the outstanding properties of graphene for transparent electrodes [17]. Synthesis or deriving process of graphene is one of the most important issues in electrode fabrication. Several methods have been reported [18]. Currently, graphene films prepared by chemical vapor deposition (CVD) method have been recognized as the most promising materials for flexible transparent electrode. However, owing to the high resistance value of graphene films, the stacked multilayer graphene films are required for electrodes, which are fabricated by complicated multiple growth and transfer processes [15]. Combining graphene with other conductor to achieve transparent flexible electrodes is an effective way, owning to its outstanding electrical and optical properties [19]. Previous works of combining CVD-grown graphene with metal microstructure has been done. For instance, the combination of Ag nanowires and graphene for use as flexible transparent electrodes has been reported [20]. While the difficulty of precisely controlling Ag nanowire distribution on film limits the further development. The above studies show that hybrid electrodes not only reduce resistance but also improve flexibility effectively. Moreover, transparent conductive oxide films are widely used in electrodes, such as ITO and aluminum-doped zinc oxide (AZO). The rigidity of these materials could be enhanced by the introduction of graphene with excellent mechanical properties. However, to our knowledge, the properties of these hybrid electrodes have not been systematically researched.

Herein, the graphene/ITO flexible hybrid transparent electrode was prepared by a two-step process: graphene film was grown on Cu foil by modified CVD method and then transferred onto ITO electrode on polyethylene terephthalate (PET) substrate. The transmission electron microscope (TEM) image, atomic force microscopy (AFM) image, and the Raman spectra of graphene films were analyzed firstly. The morphology, light transmittance, and electromechanical properties of the hybrid electrode were investigated. It is found that the gradient flux of carbon source modifies the synthesis of graphene and reduces the defect of surface structure. Moreover, the graphene layer of hybrid electrode enhances the ITO layer with excellent mechanical characteristic, and hence the electromechanical properties are improved compared to the ITO electrode.

## Methods

The graphene film was synthesized using the CVD method similar to procedures reported previously [21–23]. According to the growth kinetics and reaction mechanisms, the flow rate of precursor gas is vital for synthesis of high-quality graphene films [24, 25]. Herein, a gradient flux of the methane was developed to achieve high-quality graphene films in the growth. Briefly, a 25-μm-thick Cu foil was inserted into a 25-mm quartz tube in a tube furnace and heated to 1024 °C with 16 sccm H_2_ (pressure ~ 150 mTorr) flows. After reaching 1024 °C, the sample was left in H_2_ for 15 min and then a gradient flux of methane (CH_4_) was added to the tube for another 15 min (10 sccm for 5 min, 20 sccm for 5 min, 33 sccm for 5 min). After that, the furnace was turned off to cool down the chamber with both CH_4_ and H_2_ presence. The CH_4_ pump was turned off when the furnace temperature reached 700 °C. Then, the sample cooled down to room temperature in the presence of H_2_ continually. Meanwhile, another graphene films were prepared by the same process with constant methane flux at annealing process of 1024 °C.

The PET substrate with ITO films (ITO/PET films, thickness = 0.125 mm) were purchased from Zhuhai Kaivo Optoelectronic Technology Co., Ltd. The graphene film was transferred to ITO/PET film by means of reported reference [26-28]. Briefly, the synthesized graphene film on Cu foil was coated with the poly (methyl methacrylate) (PMMA) solution. After totally bake, PMMA was solidified to the membrane upon the graphene layer. The underlying Cu foil of hybrid film is chemically etched in the ammonium persulfate solution. Completing the etching of Cu foil, the PMMA/graphene stack was transferred to the top of the ITO/PET film and the sacrificial PMMA is removed to form the final hybrid transparent electrode.

The graphene films were characterized by Raman spectroscopy (Confocal Raman Microspectroscopy-Renishaw RM-1000) with an Ar^+^ laser excitation wavelength of 514.5 nm. The laser beam was focused onto the sample through an ×50 objective lens. The Raman spectra were collected in the frequency range of 1000 to 3000 cm^−1^ with an acquisition time of 10 s. The morphology of hybrid electrodes of graphene/ITO on PET substrate was analyzed by scanning electron microscope (CARL ZEISS-∑IGMA/VP) and eclipse inverted microscope (Nikon-TE2000U). TEM imaging was carried out by JEM-2100 (HR) with 200-kV acceleration voltage and LaB_6_ emitter. AFM imaging was conducted with tapping mode on Multimode 8 SPM system (Bruker Inc.) to test the morphology and thickness. The optical transmittance of the samples was investigated by using X-Rite spectrometer (Coloreye 7000A). The sheet resistance and other electrical data were measured by four-probe meter (KDY-1), Hall effect measurement system (Lakeshore 7704A), and LCR meter (Agilent U1731B). The mechanical force in the stretching test was conducted by an electromechanical universal testing machine (MTS, CMT-8502).

## Results and Discussion

**Fig. 1:**
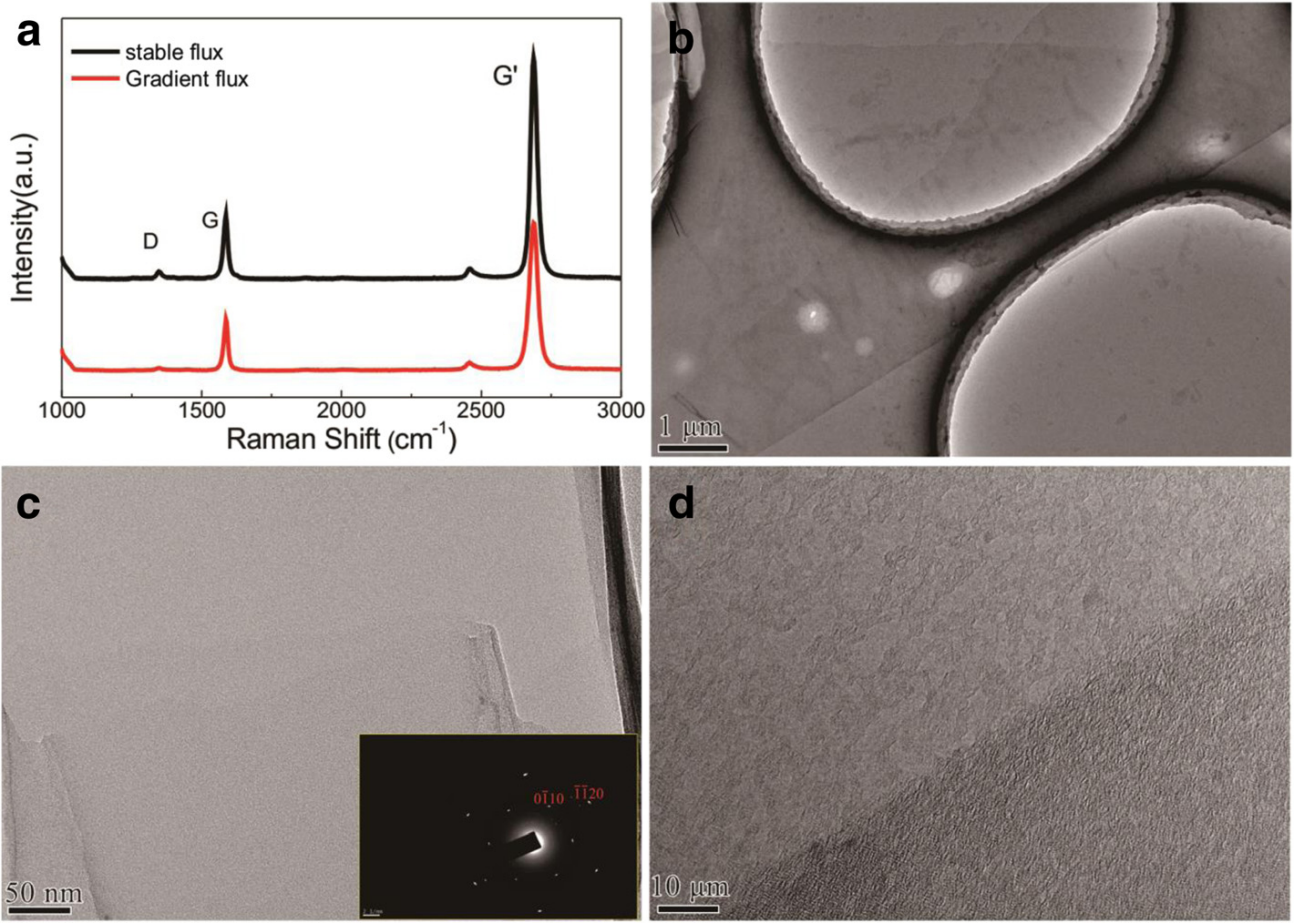
**a** Raman spectra for graphene films transferred to silicon substrate. Each *curve* shows the D peak, G peak, and 2D peak of graphene fabricated under different methane flux. **b** TEM image of graphene film with partially folded area on holey carbon grids. **c** High magnification of graphene film (TEM image); *inset images* show the SAED pattern. **d** TEM image of graphene edge

The Raman spectroscopy was used to demonstrate the composition of graphene film. Figure 1a shows the Raman spectra of graphene films with different methane flux. We believe that the way of introducing methane plays a role in decreasing defects. Thus, we compared the difference between the stable flux and gradient flux. The consistent gross of carbon source makes for eliminating the effect of other factors. The Raman spectra show three peaks located at about 1350, 1580, and 2687 cm^−1^ representing the D, G, and G′ modes of representative graphene vibration, respectively. The obvious G peak and sharp G′ peak indicate the formation of graphene. The *I*_G′_/*I*_G_ ratio for two samples is greater than two (red curve, *I*_G′_/*I*_G_ = 2.9 and black curve, *I*_G′_/*I*_G_ = 3.58), which confirms monolayer graphene film. Meanwhile, the G′ bands with the full width at half maximum around 30 cm^−1^ demonstrate the uniformity of monolayer graphene film [18]. The weak D peak is attributed to the presence of the structure defects and disorders in graphene [18, 19]. The red curve shows a relative weak D peak with a 0.03 *I*_D_/*I*_G_ ratio, comparing the black curve of 0.11. It means that the gradient flux of methane in the process of graphene growth is beneficial to decrease the defects and improve the quality of graphene.

Figure 1b–d shows the TEM images of graphene prepared with holey carbon grids with different magnification and position. Figure 1b shows the typical TEM image of graphene grown with gradient flux by CVD method. Besides, Fig. 1c shows high resolution and magnification of graphene and the inset selected area electron diffraction pattern shows hexagonal patterns, which indicates the crystallization. Figure 1d depicts the edge of graphene film. The surface topography seems smooth.

**Fig. 2:**
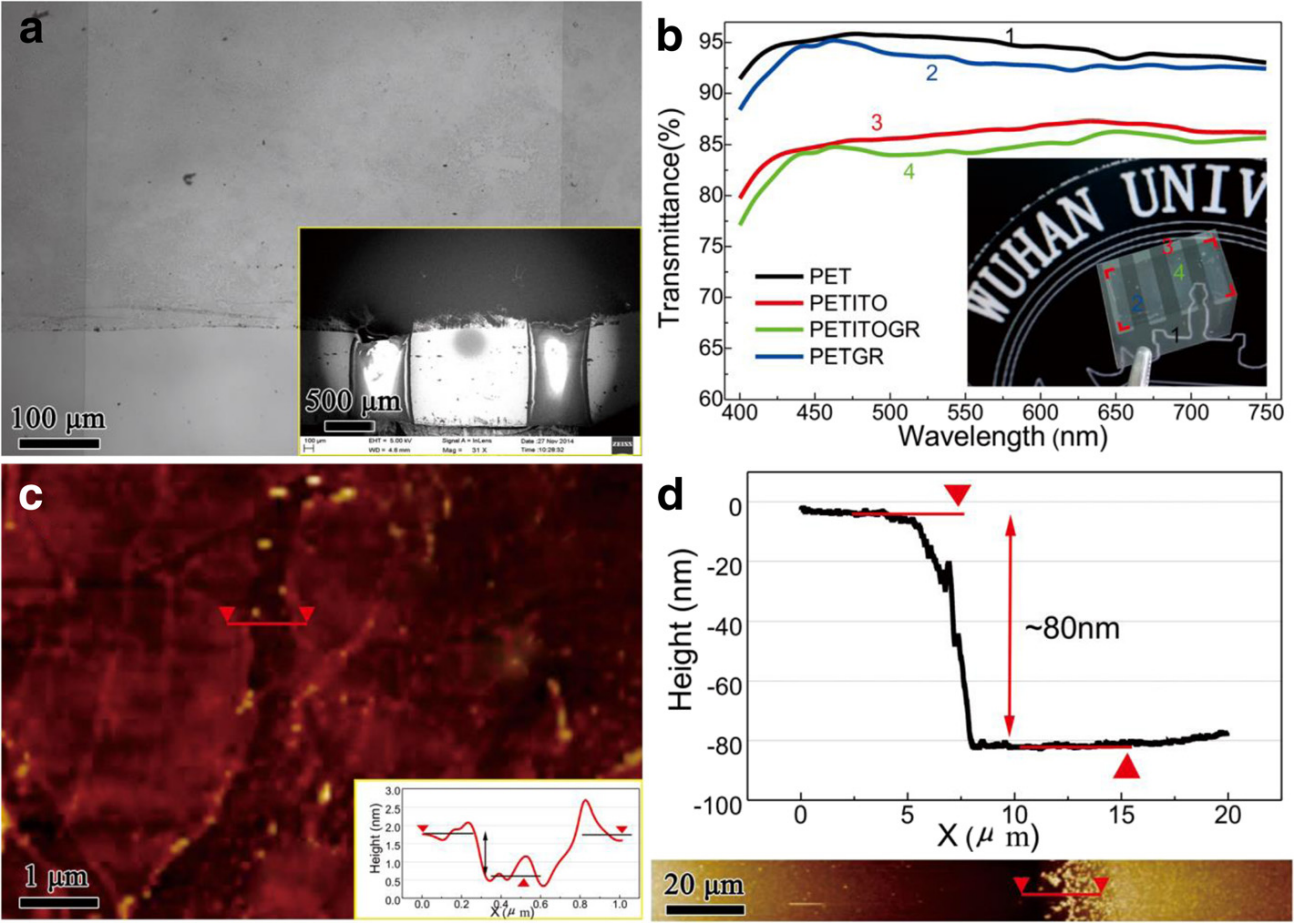
**a** Optical image of graphene/ITO hybrid electrode. *Inset SEM image* shows the surface structure of ITO bridges on *left* and *right sides* and the graphene film upon them. **b** Transmission spectra for PET substrate, ITO/PET film, graphene/ITO hybrid film on PET substrate, and the graphene/PET film. *Inset photograph* shows large domain of graphene upon ITO bridge on PET substrate. **c** AFM image of partial zone of graphene film; *red arrow* marks the distance of step area. *Inset height change image* of graphene edge marked in *red arrow*. **d** ITO edge step height measured by AFM image. *Inset image* shows the AFM image of ITO step and the marked distance of step area

The transferred graphene over the ITO electrode on PET substrate was observed by scanning electron microscope (SEM) image and optical image, as shown in Fig. 2a. The optical image shows that the graphene was well transferred on the ITO electrode in large size. In spite of the edge of the graphene film being slightly contaminated, the graphene layer is uniform, continuous, and clean. The inset SEM image shows that there are many cracks in the edge of the graphene film caused by tailor during the transfer process. In the meantime, the interior dark area proves the uniformity and the conductivity of the sample. Figure 2b shows the optical transmittance spectra of the samples on PET substrate. The optical transmittance of film samples should remove the substrate effect. We adopted the PET subtract, the graphene/PET, and the ITO/PET light absorbance to calculate the final hybrid transmittance. The figure indicates that all samples exhibit stable and high transparency property. When the effect of PET substrate absorption (~4.68 % at 550 nm) is taken into account, the transmittance of graphene film, ITO film, and graphene/ITO hybrid film wavelengths are calculated to be 97.57, 90.44, and 88.25 %, respectively. The above results show that the graphene/ITO hybrid film is enough for most transparent conducting electrode-based applications.

The sheet resistance of all samples was measured by four-probe meter and Hall effect measurement system. Other measured results were listed in Table 1. The In spot was used as test electrode for ITO and graphene/ITO film resistance testing. In our work, the sheet resistance of graphene film is ~440.34 Ω/sq, which is lower than the reported value [23], and demonstrates that the high-quality graphene film has grown in a gradient flux of methane. The sheet resistance of graphene/ITO hybrid electrode (78.34 Ω/sq) is decreased by only 2.4 % compared with the ITO electrode (76.46 Ω/sq). The conductivity of ITO can be effectively improved by introducing graphene as a hybrid electrode. We further analyzed the thickness of ITO and graphene, which is shown in Fig. 2c, d and its inset images show the height change. Figure 2c shows the detail of the AFM image of graphene boundary on silicon substrate. The graphene thickness is 0.828 ~ 1.06 nm and the roughness turns out to be ~0.93 nm. However, the ITO step shows the thickness of ~80 nm and roughness is ~2.33 nm, which is much bigger than the 2D graphene material. Besides, we added a new layer of ITO on original ITO sheet by magnetron sputtering. The thickness of the new ITO layer is close to the graphene layer. Comparing the hybrid graphene/ITO film with the added sputtering ITO film, the sheet resistance and surface carrier concentration of hybrid graphene/ITO film are better than the other. The brilliant performance in carrier mobility of graphene enhances the ITO layer mobility. It may perform better in carrier mobility by adding more layers of graphene onto ITO.

**Table 1:** The measured electrical data of the conductive electrode test in Hall effect measurement system

	Sheet resistance (Ω/sq)	Surface carrier concentration (cm^−2^)	Carrier mobility (cm^2^/V s)
ITO film	78.34	2.295 × 10^15^	32.01
Graphene	440.34	5.37 × 10^13^	1.403 × 10^4^
Graphene/ITO	76.46	2.551 × 10^15^	56.35
ITO/ITO	78.25	2.366 × 10^15^	32.71

**Fig. 3:**
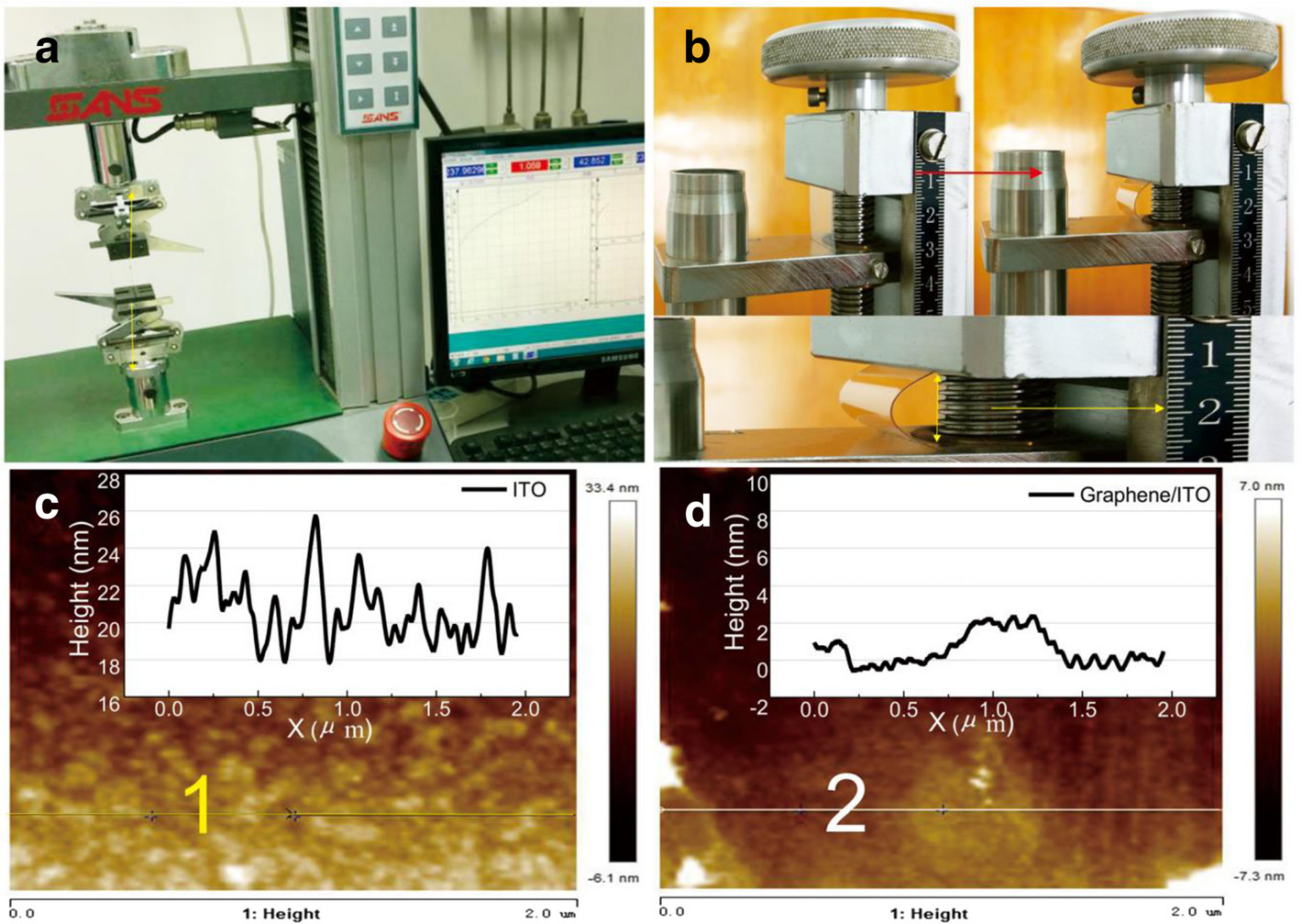
**a** The stretch process controlled by computer sets. **b** The bending process controlled by screw force. The *bottom picture* shows the bending film with the radius obtained by right scale. **c** AFM topography of the ITO film after bending process for 10 times. *Inset image* shows cross-section analysis along the *lines* shown in AFM image. **d** AFM topography of the graphene/ITO hybrid film after bending process for 10 times. *Inset image* shows cross-section analysis along the *lines* shown in AFM image

To investigate the electromechanical properties of graphene/ITO hybrid electrode, bending and stretching tests were performed. Experiments were repeated three times for each group above. Figure 3a, b illustrates the process of mechanical strain and bending. The mechanical strength was conducted by computer and screw control, which support stable mechanical and electrical change. Figure 3c, d shows AFM images illustrating the surface difference between the ITO electrode and ITO/graphene hybrid electrode after bending process. The conductive sheets with the lateral size up to 2 μm and different undulations were observed. The ITO surface is much rougher than the hybrid films. They show the general texture being consistent with what is seen in the Fig. 3b, c inset profile image. The profile direction is vertical to the bending direction. After bending, the height roughness of ITO surface changes to 8.75 nm. While the graphene film shows a narrow change of 2.84 nm. The relative undulations in lateral dimension sheets are attributed to excessive bending process on brittle ITO.

**Fig. 4:**
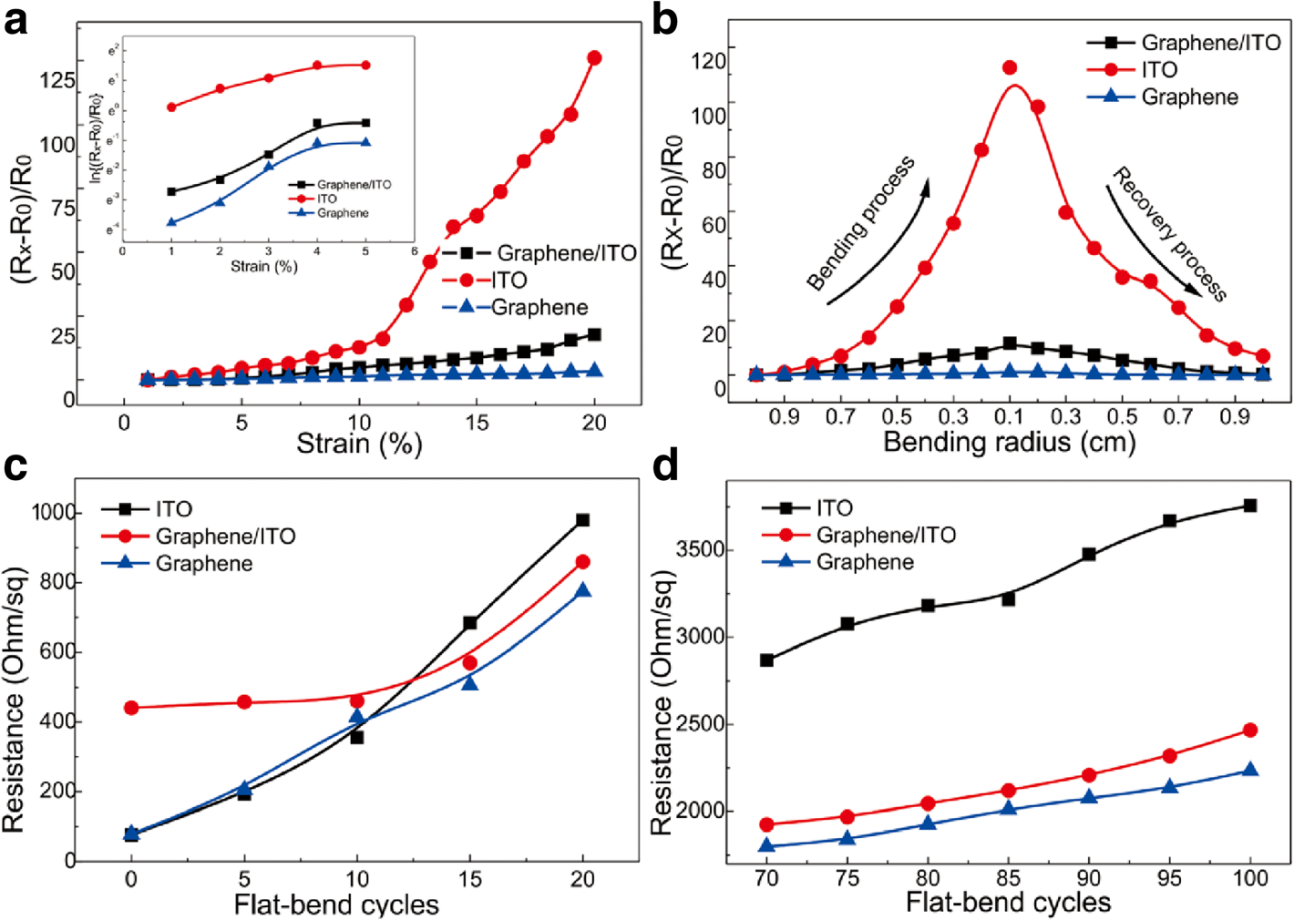
Electromechanical properties of three kinds of samples. **a** Resistance change trend versus stretch strain; *inset image* shows the detail data in a log scale. **b** Resistance change trend versus bending radius. **c** Sheet resistance change versus bending cyclic numbers between 0 and 20. Radius changed from flat to 0.1 cm. **d** Sheet resistance change versus bending cyclic numbers between 70 and 100. Radius changed from flat to 0.1 cm

In real time while observing the electrical change under stretch and bending process, we made lap joints (in electrodes) on the edge of sheet samples. The connector of LCR meter then bit the joint while the mechanical force conducted on samples. We recorded the real-time resistance data and calculated the value of △*R*/*R*_0_ to evaluate the trend. Among them, the *R*_0_ stands for the original and flat sample resistance with dimension of 1 × 1 cm^2^ and △*R* refers to the resistance change compared to *R*_0_. Figure 4a shows the mean change value in △*R*/*R*_0_ of the samples as a function of the applied tensile strain. The resistance of samples rises up as tensile strain increases. Before the 5 % tensile strain, the value of the three electrode △*R*/*R*_0_ is nearly square. For clearly distinguishing the difference, the data was shown in a log scale in the inset image. After 10 % tensile strain, the brittle property of ITO is evident. Unlike the ITO electrode, the graphene electrode could resist up to 20 % strain with △*R*/*R*_0_ of ~3.28 compared with that of ~125.91 for ITO at the same strain. Likewise, the hybrid graphene/ITO electrode is well performed with △*R*/*R*_0_ of ~17.78. The result demonstrates the pronounced advantage of graphene in protecting ITO brittle failure to some extent. It could be possible to take use of graphene hybrid conductors in flexible transparent electrode.

A comparative experiment for resistance change trend measurements was also performed under stable bending process at different curvature. The bending radius was measured by the scale, which is shown in Fig. 4b. When the bending radius is 0.1 cm by adjusting the screw smoothly, the value of ITO electrode rises to ~112.51 while that of graphene/ITO hybrid electrodes to ~1.11 and hybrid film to ~11.65. When the electrodes recover to be flat, the value of ITO electrode is back to be ~6.92, while the graphene/ITO hybrid electrode is back to ~0.27. Such result shows the benefit of graphene in terms of mechanical flexibility over ITO electrode, which is favorable to ITO. Figure 4c presents the sheet resistance of the samples with the same dimension, as they are bent multiple times at the bending radius of 0.1 cm. The smooth force makes sure of the secular change of PET substrate and decreases the test error. After 5 cycles of bending test, the sheet resistance was measured by four-probe meter. Original sheet resistance value of graphene electrode is bigger than the hybrid electrode and ITO. Such situation changed after 15 cycles of bending. We obtained a sheet resistance of ITO with 683.98 Ω/sq, graphene with 570.24 Ω/sq, and hybrid electrode with 506.37 Ω/sq. Currently, the three kinds of samples share similar resistance value. As more cycles of bending was made, the sheet resistance of ITO changed greatly and rise to ~3757.2 Ω/sq finally.

## Conclusions

In summary, this work demonstrates a feasible two-step process method to employ the CVD graphene in fabricating the graphene/ITO flexible hybrid transparent electrode. With this method, as-fabricated graphene shows lower defect and improves the mechanical stability of single ITO electrode. Such hybrid electrode makes a significant advancement toward traditional transparent flexible film both in electrical and optical properties. The hybrid method meets the criteria of low cost, conductivity, and mechanical stability. The graphene/ITO hybrid film as a flexible transparent electrode has important implications for future devices.
